# The blood-brain tumor barrier as a spatial immune checkpoint in glioblastoma: leukocyte entry, perivascular retention, and therapeutic implications

**DOI:** 10.3389/fimmu.2026.1957858

**Published:** 2026-10-05

**Authors:** Fangbo Lin

**Affiliations:** Rehabilitation Medicine Department, The Affiliated Changsha Hospital of Xiangya School of Medicine, Central South University, Changsha, China

**Keywords:** blood-brain tumor barrier, glioblastoma, immunotherapy resistance, leukocyte trafficking, neurovascular unit, perivascular myeloid niches, spatial immune checkpoint, tumor-associated macrophages

## Abstract

Glioblastoma (GBM) combines heterogeneous vascular dysfunction with a predominantly myeloid immune landscape. This Mini Review focuses on a specific problem: vascular permeability is not equivalent to productive leukocyte access. Entry requires endothelial capture, chemokine-guided arrest, transendothelial migration, release from perivascular sites, intratumoral dispersion, target recognition, and sustained effector function. Human tissue and spatial studies show regionally distinct neurovascular and myeloid states, whereas much of the causal evidence for trafficking and retention remains preclinical. We therefore distinguish published observations from a hypothesis-generating model in which the blood-brain tumor barrier (BBTB) and perivascular myeloid niche can act as coupled but separable checkpoints. The main conceptual advance is not the already recognized heterogeneity of the BBTB, but the argument that leakage-based imaging should not be used as a surrogate for immune-cell entry and that each trafficking stage requires its own pharmacodynamic readout. We summarize regional BBTB states, differential lymphoid and myeloid access, and selected strategies—vascular normalization, myeloid reprogramming, local cell delivery, and focused ultrasound—while emphasizing their evidence limits. Clinical translation will require spatially registered human sampling, explicit model provenance, and tests that can reject individual components of the framework.

## 1 Introduction

Glioblastoma (GBM) remains difficult to control despite maximal resection, radiotherapy, and temozolomide (1, 2). In recurrent GBM, nivolumab did not improve overall survival over bevacizumab in CheckMate 143 (3). Small neoadjuvant anti-PD-1 studies nevertheless demonstrated intratumoral immune and transcriptional changes, indicating biological activity without establishing broad clinical efficacy (4, 5). The contrast between measurable immune activation and limited population-level benefit suggests that drug class alone is an incomplete explanation. It also motivates a closer examination of when circulating or transferred immune cells can reach tumor tissue and which step fails after arrival.

Clinical immunotherapy studies illustrate the narrower interpretive problem. An intrathecal bivalent CAR-T trial reported early decreases in gadolinium enhancement or tumor size, and an intraventricular CARv3-TEAM-E study reported rapid radiographic regression; both used MRI change as an early biological-activity or response signal (6, 7). Neither study claimed that enhancement directly measured immune-cell access. We therefore do not present this as a universal field assumption. Regional BBTB heterogeneity is established (8, 9), and this review treats the BBTB as a multicellular interface that influences successive trafficking stages. The focused point is that contrast enhancement and tracer leakage report solute behavior or radiographic change, not sequential leukocyte capture, diapedesis, distribution, and function. These stages should be measured separately rather than collapsed into the label “immune infiltration.

To delimit this narrative Mini Review, PubMed was searched through July 2026 for peer-reviewed English-language studies using combinations of “glioblastoma,” “blood-brain barrier” or “blood-brain tumor barrier,” “endothelium,” “leukocyte trafficking,” “spatial,” “myeloid,” “CAR T,” and “focused ultrasound.” Recent human GBM tissue, single-cell/spatial, and clinical studies (principally 2018-2026) were prioritized; older landmark studies and experimental GBM models were retained when needed for mechanism. Reference lists of key reviews were also screened. This was a targeted narrative search, not a systematic review or quantitative synthesis. Throughout, evidence is identified as human associative, human interventional, preclinical mechanistic, or authorial hypothesis.

## 2 Regional BBTB heterogeneity and evidence provenance

The normal blood-brain barrier depends on specialized endothelial transport, tight and adherens junctions, pericytes, astrocytic endfeet, and basement membrane. In GBM these elements are remodeled asynchronously: junctional leakage, transcellular transport, perfusion, endothelial inflammatory activation, and leukocyte adhesion can therefore diverge within the same lesion (8, 9). Permeability and leukocyte recruitment are especially important to separate: a vessel may pass contrast or small solutes yet remain poorly perfused, express insufficient luminal adhesion molecules, or fail to present the chemokines needed for firm arrest. Conversely, endothelial activation need not require gross barrier rupture. An enhancing core, infiltrative margin, and hypoxic vascular niche should therefore not be treated as one vascular state.

Experimental glioma studies show that invading tumor cells can disrupt astrocyte-vascular coupling (10). A human-cell microfluidic BBTB model reproduced selected permeability changes, tumor-endothelial interactions, and immune-cell binding, but it did not establish complete leukocyte diapedesis or productive trafficking in patients (11). Analysis of human GBM demonstrated cell-specific transporter remodeling without uniform loss of barrier features (12). Comparisons among such models require reporting endothelial and tumor-cell origin, matrix composition, molecular size, shear conditions, and whether the assay measures solute flux, cellular binding, passage, or both.

Pericyte and matrix states add context rather than a single directional effect. In experimental GBM models, pericyte depletion accelerated tumor progression and reshaped endothelial and immune states (13), supporting the view that some pericyte programs may restrain tumor growth and arguing against indiscriminate depletion. Experimental studies implicate disrupted astrocyte-vascular coupling and pericyte state as modifiers of the endothelial interface (10, 13). Direct human measurements of leukocyte migration through these states remain limited. Human spatial atlases associate vascular, hypoxic, invasive, and immune neighborhoods with local tumor programs (14, 15), while longitudinal human profiling suggests that therapy reorganizes this architecture (16). These studies establish spatial association; most causal links between a specific vascular state and leukocyte passage still require functional validation. **Table 1** summarizes the resulting evidence map.

**Table 1:** Analytical framework for BBTB trafficking determinants, evidence provenance, limitations, and discriminating experiments in glioblastoma.

Domain/determinant	Species/model system	Evidence class	Key limitation	Critical or discriminating experiment	Key refs
Endothelium and junctions	Human GBM tissue; human-cell BBTB models	Human associative; *in vitro* mechanistic	Leakage or bulk ICAM-1 does not establish luminal adhesion or diapedesis.	Under flow, measure solute flux, luminal ICAM-1, cell arrest, completed passage, and extravascular position in the same vessel segment.	(8, 11)
Pericytes, astrocytes, and matrix	Experimental glioma; human spatial tissue	Preclinical causal; human associative	Effects vary by cell state and region; direct human migration data are scarce.	Perturb one compartment under flow, quantify leukocyte passage and post-endothelial dispersion, and benchmark states to human tissue.	(10, 13)
Infiltrative, enhancing, and hypoxic regions	Human MRI-guided tissue; spatial atlases	Human associative	Spatial matching and longitudinal sampling are limited; enhancement is not entry.	Co-register MRI-defined regions with intravascular/extravascular immune localization and vessel-distance analysis from the same coordinates.	(14, 16)
Myeloid entry and perivascular localization	Human single-cell/spatial studies; murine recruitment models	Human associative; preclinical causal	Entry route, lineage, and dwell time are rarely resolved.	Use lineage-resolved time courses before and after a recruitment or niche perturbation; show altered retention without altered entry.	(17, 18)
Lymphocyte availability, entry, and function	Patient specimens/cohorts; intracranial-tumor models	Human associative; preclinical mechanistic	Cell density does not prove extravascular position or function; systemic sequestration confounds inference.	Pair blood/marrow availability with tissue measures of capture, extravasation, dispersion, tumor contact, and effector function.	(19, 20)
Recurrence, radiotherapy, and dexamethasone	Longitudinal human tissue; clinical cohorts	Human associative	Timing, dose, treatment, and biopsy location confound comparison.	Obtain spatially matched longitudinal samples with treatment intervals and steroid/antiangiogenic exposure prespecified.	(16, 21)
Vascular normalization or focused ultrasound	Human vascular/BBB-opening trials; murine FUS immune models	Human interventional; preclinical mechanistic	Permeability or drug delivery does not establish immune-cell trafficking.	Time perfusion/permeability with luminal adhesion, cell passage, dispersion, and function before and after intervention.	(22, 23)
Local CAR-T delivery	Small early-phase human trials	Human interventional feasibility	Uncontrolled samples and radiographic endpoints do not resolve whole-tumor dispersion or durability.	Compare labeled local versus systemic cells, or serially track distribution and function beyond the infusion site.	(6, 7)

## 3 The BBTB as a selective gateway for leukocyte trafficking

### 3.1 Endothelial adhesion and chemokine matching

Productive leukocyte entry requires endothelial capture, adhesion, and transendothelial migration under flow; junctional opening alone does not establish these steps (24). Reviews of the BBTB describe marked regional variation in vascular integrity and permeability across GBM (8, 9). That evidence does not establish that an individual leaky vessel segment also supports inflammatory leukocyte capture. In primary human brain microvessel endothelial monolayers, ICAM-1 blockade reduced T-cell adhesion and almost abolished migration across activated endothelium, supporting a functional role for endothelial ICAM-1 (25). However, bulk tissue ICAM-1 abundance does not establish luminal recruitment competence because the signal may derive from non-endothelial cells or non-luminal endothelial surfaces. Perfusion, luminal adhesion competence, and completed diapedesis should therefore be measured separately from leakage, although direct patient-level proof remains limited.

Chemokine-receptor matching can select the leukocytes available to a vessel segment. In GBM, reported axes include CXCL9/10-CXCR3, CCL2-CCR2, and CXCL12-CXCR4, although their effects vary by cellular source and disease context (26). For vascular recruitment, the relevant variable is not simply total ligand abundance but presentation on the appropriate luminal segment when receptor-bearing cells are available (24). Human single-cell atlases define disease-specific immune states (17, 27), but transcript abundance or bulk chemokine concentration does not demonstrate luminal presentation under flow. Claims about productive recruitment therefore require spatial endothelial localization, receptor assessment on circulating cells, and mapping of extravascular cells beyond the immediate perivascular compartment.

### 3.2 Differential entry of lymphoid and myeloid cells

Single-cell analysis of experimental glioma resolves resident microglia, infiltrating monocytes/macrophages, and border-associated macrophage states (28). Human tumor and model data further show that macrophage-derived signals can drive mesenchymal-like tumor programs (29). A recent human study identified distinct myeloid-derived suppressor-cell populations with different spatial and metabolic associations (30). Together, these studies support myeloid heterogeneity and spatial association, but they do not establish the vascular route or rate of entry. They also argue against reducing GBM myeloid biology to a single M1/M2 axis, because lineage and local tumor state can generate overlapping suppressive, inflammatory, and repair programs. Causal recruitment mechanisms remain derived largely from murine glioma, *in vitro*, or ex vivo systems, and mechanistic statements below are labeled accordingly.

Lymphoid limitation begins before the vessel. Intracranial-tumor models supported by patient observations link surface S1PR1 loss to bone-marrow T-cell sequestration (19), and human GBM specimens show severe T-cell exhaustion (20). In clinical cohorts, concurrent dexamethasone is associated with reduced benefit from immune checkpoint blockade (21). These processes operate at different levels: sequestration reduces the circulating pool, endothelial failure limits capture, and exhaustion constrains cells that enter. Dexamethasone can affect both systemic availability and local vascular-immune readouts, so dose and timing should be treated as biological covariates rather than background medication. Thus low intratumoral T-cell density can reflect limited systemic availability, failed endothelial capture, poor tissue dispersion, or functional impairment. Bulk myeloid abundance is therefore a poor proxy for lymphoid access or infiltration. Myeloid, NK, and CAR-T populations differ in systemic availability, receptor and adhesion programs, entry routes, and persistence; each requires cell-specific spatial assessment.

### 3.3 Regional and treatment-dependent trafficking states

Trafficking states also depend on biopsy location and treatment history. Infiltrative margins may retain barrier features, whereas enhancing and hypoxic regions contain distinct vascular and immune neighborhoods (9, 14). Paired primary and recurrent human specimens show post-treatment reorganization of vascular, astrocytic, and lymphocyte-associated spatial relationships (16). Because that small longitudinal cohort could not isolate the contributions of radiotherapy, temozolomide, recurrence, antiangiogenic exposure, and glucocorticoids, recurrent tumors should not be assumed to reproduce the trafficking state of untreated GBM. Treatment-dependent mechanisms should therefore be stated cautiously.

Cross-study comparisons should report biopsy coordinates, disease stage, interval from radiotherapy, steroid dose, and antiangiogenic exposure (8, 16). Recommended spatial endpoints are intravascular versus extravascular localization, endothelial adhesion-molecule expression, distance from the nearest vessel, radial dispersion, tumor-cell contact, and effector markers. Vessel identity should also be recorded because co-opted capillaries, angiogenic microvessels, and post-radiation vessels may present different adhesion and basement-membrane conditions. Whole-tumor leukocyte density alone can misclassify cells that remain intravascular or accumulate immediately outside the vessel as productive infiltration.

## 4 Perivascular myeloid niches: evidence and hypothesis

### 4.1 Cellular composition and spatial organization

After extravasation, leukocytes encounter a regionally variable perivascular compartment containing endothelial cells, pericytes, extracellular matrix, tumor cells, resident microglia, and recruited macrophages. Human spatial studies define regionally organized neighborhoods (14, 15), while human single-cell studies distinguish immune and suppressive myeloid states (17, 30). Assignment of specific suppressive functions or migration kinetics generally comes from experimental models. Perivascular macrophage-like cells may represent resident, recruited, or convergent states, and morphology alone cannot resolve their origin. Cell identity should therefore be supported by transcriptomic, anatomical, and, where possible, epigenetic evidence. This provenance matters therapeutically because depletion or reprogramming may affect resident microglia and blood-derived macrophages differently.

Single-cell analysis of experimental glioma resolves distinct spatial distributions of microglia, infiltrating monocytes/macrophages, and border-associated macrophages (28). Paired human tumors show that treatment reorganizes vascular, astrocytic, and lymphocyte-associated spatial relationships (16). Neither static sections nor dissociated single-cell data resolve direction or dwell time, and marker convergence can obscure lineage. The niche is best treated as a dynamic combination of origin, oxygenation, vascular phenotype, matrix, and therapeutic exposure rather than a fixed macrophage subtype.

### 4.2 Mechanisms of leukocyte sequestration and suppression

In murine glioma, CSF1R inhibition reprogramed macrophages and delayed tumor growth (31), but compensatory signaling produced resistance (32). A separate experimental GBM study implicated CXCL12 in monocyte-derived macrophage recruitment (18). These pathways pertain to different trafficking stages. CXCL12 is relevant to recruitment and local maintenance, whereas CSF1R primarily regulates macrophage survival and state. TGF-β, IL-10, arginase-related nutrient restriction, oxidative stress, PD-L1, and adenosine signaling mainly describe functional suppression after cells have arrived; VEGF and matrix-remodeling enzymes may reshape vascular or stromal conditions but do not, by themselves, establish physical sequestration (33). Human spatial data show proximity among vascular, tumor, and myeloid programs but not causal order or a time-resolved feedback loop (14, 15). We therefore use “perivascular immune trap” only as a hypothesis. Evidence for a true trap would require completed transendothelial migration followed by prolonged perivascular dwell time, reduced radial movement and tumor-cell contact, and release after niche perturbation without a parallel change in entry. Time-resolved imaging or repeated spatial sampling linked to a perturbation would be needed; perivascular localization in a static section would not meet this criterion.

### 4.3 Consequences for immunotherapy

Checkpoint inhibition depends on available, functional tumor-reactive T cells, which are constrained by severe exhaustion in GBM (20). CAR-T cells bypass priming but remain vulnerable to antigen heterogeneity and local suppression, which are persistent barriers in glioma immunotherapy (33). Local or intraventricular delivery can bypass part of the endothelial route, but uniform parenchymal dispersion still requires direct measurement. Radiographic change can reflect focal activity, edema, or necrosis and therefore cannot establish durable trafficking. Tissue or validated cell-tracking endpoints are needed to distinguish delivery from durable functional distribution.

Entry, perivascular retention, intratumoral dispersion, target recognition, and sustained effector function should be evaluated as separate pharmacodynamic stages. Candidate readouts include endothelial ICAM-1/VCAM-1 for recruitment competence, extravascular localization for passage, vessel-distance analysis for dispersion, tumor-cell contact for recognition, and granzyme or cytokine activity for function. These measurements are most informative when obtained from spatially matched pre- and post-treatment samples and interpreted with circulating-cell availability (14, 16). A favorable upstream stage may coexist with downstream failure; a treatment that increases perivascular cells without increasing radial dispersion should not be described as having restored productive infiltration (**Figure 1**).

**Figure 1:**
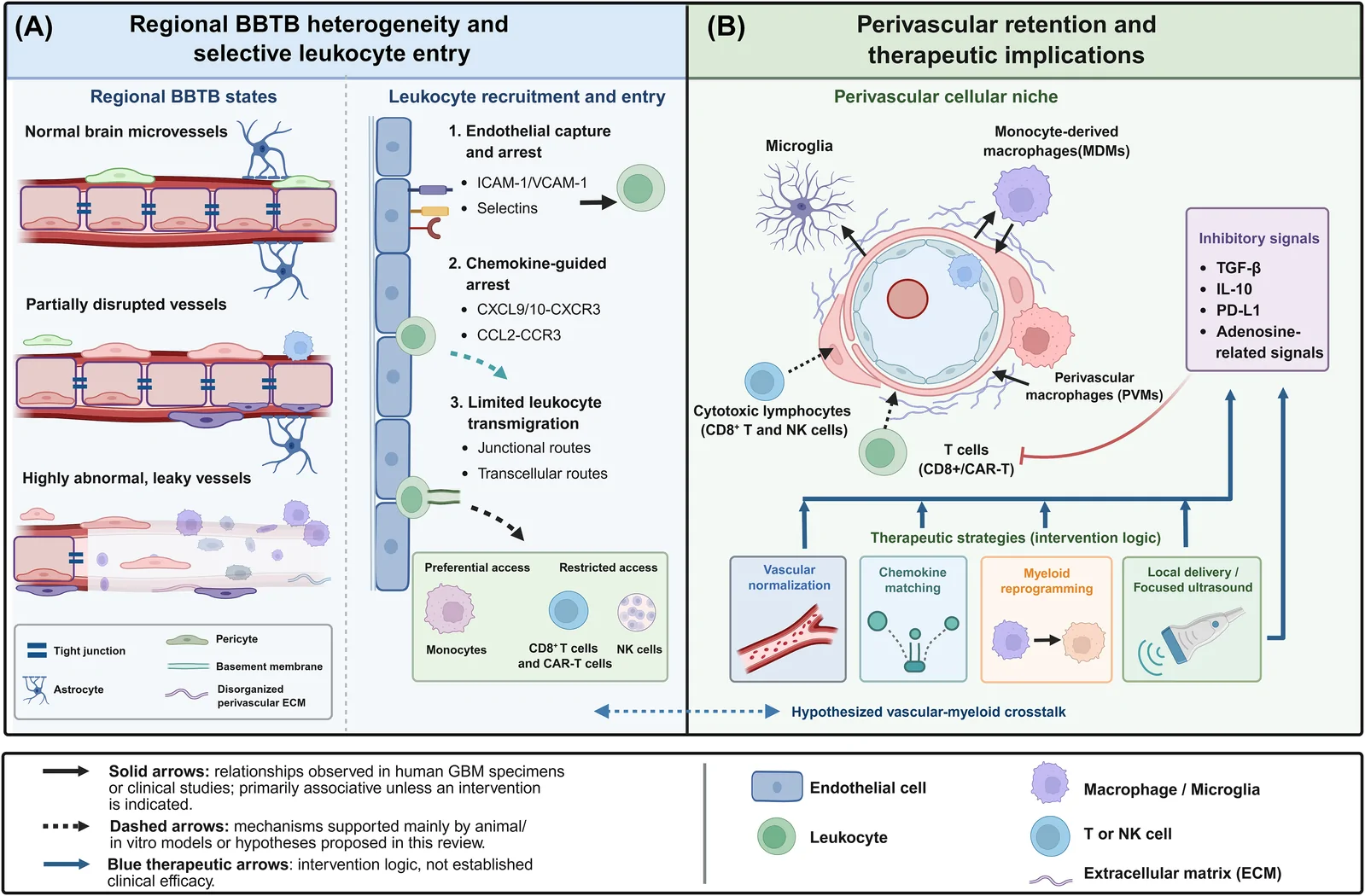
Regional BBTB heterogeneity and its effects on immune-cell trafficking in glioblastoma. **(A)** Different vascular states regulate leukocyte recruitment and entry. **(B)** The perivascular myeloid niche may restrict lymphocyte dispersion and function and represents a potential therapeutic target. Solid arrows indicate human GBM or clinical evidence, dashed arrows indicate preclinical evidence or hypotheses, and blue arrows indicate proposed therapeutic strategies. BBTB, blood-brain tumor barrier; GBM, glioblastoma.

## 5 Therapeutic implications for the neurovascular-immune interface

Vascular normalization should be distinguished from nonspecific barrier opening. In a clinical GBM study, the VEGFR inhibitor cediranib transiently improved vascular function and reduced edema (22). However, recurrent GBM specimens obtained after antiangiogenic therapy showed increased tumor-associated macrophages associated with poorer survival (34). These human findings indicate that vascular and immune responses can move in different directions; neither study proves that normalization increases productive leukocyte trafficking. The cediranib study also showed that normalization was time-dependent and reversible (22). Trials should therefore measure perfusion, permeability, endothelial activation, and immune location in parallel rather than infer one from another.

Mechanistically matched strategies could combine improved perfusion and endothelial responsiveness with chemokine-receptor matching or myeloid reprogramming. In experimental glioma, CSF1R inhibition showed both activity and adaptive resistance (31, 32), whereas a separate model implicated CXCL12 in recruited-macrophage maintenance (18). Broader myeloid interventions remain supported mainly by preclinical evidence (33) and should be selected by the dominant regional state rather than applied indiscriminately. Pharmacodynamic tissue sampling should demonstrate altered cell location and function, not only changes in circulating-cell counts. Reprogramming may be preferable to broad depletion when resident microglia retain tissue-supportive functions, but this is a therapeutic hypothesis rather than a clinically established rule. Safety assessment should also capture edema and tissue injury.

Local CAR-T delivery provides feasibility and biological-activity signals, not definitive validation of the trafficking model. In the six-patient intrathecal bivalent CAR-T study, all patients showed early reductions in enhancement or tumor size, but none met objective response criteria (6). The three-patient intraventricular CARv3-TEAM-E study reported rapid radiographic regression, with two transient responses (7). Both studies were uncontrolled, and small samples, limited durability, steroid or toxicity management, and radiographic confounding restrict inference. A single-patient IL13Rα2 report documented a 7.5-month response (35), while intravenous EGFRvIII CAR-T showed antigen loss and adaptive resistance despite tumor trafficking (36). None compared local with systemic delivery or quantified whole-tumor dispersion. The evidence therefore supports feasibility and short-term activity, not that bypassing the vascular route is sufficient for durable control.

Focused ultrasound can transiently increase barrier permeability. In a murine glioma model, it increased CAR-T-cell delivery and enhanced responses to several immunotherapies (23). A phase I/II trial evaluated repeated opening with carboplatin (37), and a phase 1 study evaluated albumin-bound paclitaxel delivery (38). An earlier first-in-human study primarily established the safety and feasibility of repeated disruption before carboplatin (39). None of these clinical studies established improved leukocyte adhesion, diapedesis, dispersion, or function. Focused ultrasound for immune-cell trafficking should therefore remain investigational and use stage-specific immune endpoints rather than permeability alone, consistent with a recent neuro-oncology synthesis (40). The acoustic exposure, vascular segment, timing relative to cell infusion, and transient inflammatory response may all affect outcome. A rise in contrast leakage or drug concentration after sonication should not be interpreted as evidence that transferred or endogenous effector cells have completed the same route.

The proposed sequence is a research roadmap, not a clinic-ready workflow. Several inputs are currently measurable: perfusion and leakage with contrast MRI, steroid and antiangiogenic exposure, circulating leukocyte availability, and, when tissue is obtained, intravascular versus extravascular location, vessel distance, tumor-cell contact, and effector markers. These measures can test whether an intervention changes a defined trafficking stage, but imaging alone cannot establish immune access. Other inputs remain investigational: leakage-independent mapping of regional BBTB state; luminal ICAM-1/VCAM-1 and chemokine presentation under physiological flow; real-time diapedesis; and serial estimates of perivascular dwell time or radial dispersion in unsampled regions. These gaps will require vascular-selective probes, cell tracking, spatially registered tissue, or improved human BBTB models. Safety monitoring must include edema, hemorrhage, neurotoxicity, and systemic inflammation. The objective is controlled productive trafficking, not maximal permeability.

## 6 Discussion: measurement and testable predictions

BBTB studies need terminology that separates permeability, endothelial recruitment competence, transendothelial migration, perivascular residence, intratumoral dispersion, recognition, and function. Prospective studies should pair imaging with spatially registered tissue, record biopsy coordinates and treatment intervals, and report glucocorticoid and antiangiogenic exposure. These minimum data would make human observations and experimental models easier to compare without treating contrast enhancement as immune access. Studies should also state whether evidence is derived from human GBM, another intracranial tumor, a murine glioma model, or an engineered *in vitro* system. This sentence-level provenance is particularly important when human spatial association is discussed alongside preclinical perturbation, because only the latter can usually establish causal direction.

Informative designs will combine single-cell or spatial profiling with multiplex imaging and three-dimensional vascular reconstruction in matched newly diagnosed and recurrent tumors. Human endothelial-pericyte-astrocyte models should incorporate flow, tumor cells, matrix, and immune cells, and should quantify each trafficking stage separately. Because lineage tracing is not available in patients, microglial versus monocyte-derived identity requires convergent molecular and anatomical evidence. Functional models should report whether they reproduce regional barrier properties and should be benchmarked against human tissue rather than treated as complete BBTB replicas. Fluid biomarkers can complement but not replace tumor localization.

The framework makes stage-specific predictions rather than one all-or-none claim. One operational prediction is that an intervention that increases endothelial adhesion competence and verified transendothelial T-cell passage will increase extravascular T-cell numbers, but may fail to improve dispersion or function when a perivascular checkpoint remains active. If repeated spatial analysis show equivalent radial dispersion and tumor-cell contact regardless of perivascular myeloid density or state, the proposed retention component would not be supported. Conversely, if improved adhesion and diapedesis consistently fail to increase extravascular cells, the endothelial checkpoint mechanism should be revised. Likewise, if increasing extravascular lymphocytes restores tumor-cell contact and effector function without altering the perivascular myeloid niche, the claim that niche reprogramming is generally required would be weakened. A downstream failure therefore challenges the corresponding stage rather than proving that vascular biology is irrelevant. This focused, testable formulation treats the coupled-checkpoint model as hypothesis-generating until validated in human GBM and avoids presenting a conceptual synthesis as an established human mechanism.
